# Knowledge, Attitude, and Practices Regarding Metabolic Syndrome Among Ambulance Support Personnel

**DOI:** 10.7759/cureus.112241

**Published:** 2026-07-07

**Authors:** Jheel R Tripathi, Suraj Kanase, Sakshi P Kavitake

**Affiliations:** 1 Department of Neuro-Physiotherapy, Krishna College of Physiotherapy, Krishna Vishwa Vidyapeeth (Deemed to be University), Karad, IND

**Keywords:** ambulance support personnel, attitude, knowledge, lifestyle modification, metabolic syndrome, occupational health, practices

## Abstract

Background

Metabolic syndrome (MetS) is a clustering of interrelated physiological disturbances, including central adiposity, hypertension, dyslipidemia, insulin resistance, and impaired glucose regulation, that collectively amplify susceptibility to cardiovascular disease and type 2 diabetes mellitus. Ambulance support personnel encounter an array of occupational stressors, including prolonged shift durations, erratic meal schedules, chronic sleep deficit, and persistent work-related stress, which may heighten their predisposition to metabolic dysfunction. Despite the indispensable role of this workforce within the emergency healthcare system, research examining their awareness and health-protective behaviors regarding metabolic syndrome remains sparse.

Methods

A Google Form-based survey was conducted among 119 ambulance support personnel working in Karad City, Maharashtra, India. All participants voluntarily completed a structured questionnaire. The questionnaire consisted of demographic information and questions assessing knowledge, attitude, and practices (KAP) regarding metabolic syndrome. Responses were collected and analyzed using descriptive statistical methods to determine the level of awareness and preventive health behaviors among the participants.

Results

Among the 119 ambulance support personnel, 58.0% demonstrated moderate knowledge regarding metabolic syndrome, 67.2% exhibited a positive attitude toward its prevention and management, and 60.5% demonstrated fair preventive practices. The mean knowledge, attitude, and practice scores were 5.8 ± 1.4, 4.9 ± 1.1, and 5.7 ± 1.8, respectively.

Conclusion

Ambulance support personnel demonstrated moderate knowledge and generally positive attitudes regarding metabolic syndrome; however, preventive practices were comparatively less satisfactory. Workplace-based health promotion programs focusing on lifestyle modification, health screening, and behavioral change strategies may help reduce metabolic risk in this population.

## Introduction

Metabolic syndrome (MetS) constitutes a convergence of interrelated physiological abnormalities, encompassing central adiposity, arterial hypertension, dyslipidemia, insulin resistance, and dysregulated glucose homeostasis [1]. Collectively, these conditions increase the risk of cardiovascular disease, type 2 diabetes mellitus, and premature mortality [2,3]. Over recent decades, MetS has emerged as a formidable public health challenge globally, driven by its accelerating prevalence and tight association with a broad spectrum of non-communicable diseases [4,5].

The worldwide burden of metabolic syndrome has grown considerably across both industrialized and developing nations [4,5]. Rapid urbanization, sedentary behavior, suboptimal dietary habits, and work-related stress are among the principal contributors to this upward trend [5]. In India, socioeconomic transitions and evolving lifestyle patterns have catalyzed a significant rise in the prevalence of obesity, hypertension, diabetes, and allied cardiometabolic disorders [6]. Consequently, MetS has become a foremost driver of the country’s non-communicable disease burden [5,6].

The etiological contributors to MetS encompass both modifiable and non-modifiable elements. Non-modifiable determinants include advancing age, genetic susceptibility, and a positive family history [7,8]. Conversely, lifestyle-related factors, physical inactivity, excess body weight, poor dietary quality, tobacco use, alcohol consumption, chronic psychological stress, and insufficient sleep, play pivotal roles in the onset and progression of the condition [8,9]. Given the amenability of these factors to behavioral intervention, preventive strategies centered on lifestyle modification have assumed increasing importance in both public health practice and clinical management [10].

Habitual physical exercise, the maintenance of a healthy body weight, adherence to a nutritionally balanced diet, effective stress regulation, and restorative sleep have each demonstrated efficacy in reducing the risk of MetS and its downstream complications [10]. Disseminating the awareness of these preventive strategies is therefore indispensable for fostering health-promoting behaviors and alleviating the chronic disease burden. Knowledge of risk factors and protective measures generally shapes an individual’s health-related attitudes, which in turn influence the likelihood of translating awareness into consistent lifestyle practice [11].

Healthcare professionals may be at an increased risk of MetS because of the demanding nature of their occupational environment [12]. Extended working hours, rotating shift schedules, night duties, work-related stress, erratic meal patterns, and disrupted sleep collectively impose significant strain on metabolic health [13,14]. Comparative investigations have documented significantly higher rates of obesity, hypertension, and metabolic abnormalities among shift-working healthcare personnel relative to those maintaining conventional work schedules [14,15].

Operational requirements frequently include sustained vigilance across irregular duty schedules, exposure to distressing events, and severely curtailed opportunities for regular nutrition or adequate rest [16]. Within the healthcare workforce, ambulance support personnel constitute a distinctive occupational subgroup confronted with a particularly challenging array of physical and psychological demands. Their responsibilities encompass emergency patient care, urgent patient transportation, and rapid deployment in unpredictable, high-acuity circumstances [17]. These working conditions may foster maladaptive behavioral patterns and amplify cardiometabolic vulnerability [14,17].

Shift work and nocturnal duties, both defining features of ambulance services, carry well-documented associations with metabolic disruption [14,17]. Circadian rhythm dysregulation arising from nonstandard work schedules has been causally linked to obesity, insulin resistance, hypertension, and unfavorable lipid profiles [15]. Superimposed occupational stress and chronic sleep restriction may further potentiate the risk of developing MetS [13,16]. Despite these recognized occupational hazards, investigations specifically examining awareness levels and preventive health behaviors among ambulance support personnel remain limited in number [18].

Knowledge, attitude, and practice (KAP) assessments are widely used to evaluate awareness and preventive health behaviors related to chronic diseases. They help identify gaps between awareness and actual health practices, thereby guiding the development of targeted health promotion strategies.

Prior research has established that individuals may possess a solid theoretical understanding of metabolic syndrome while consistently failing to enact corresponding lifestyle changes [12]. This divergence between knowledge and action, commonly termed the knowledge-practice gap, represents a significant obstacle in preventive healthcare [12]. Characterizing this gap is foundational to the design of effective health education curricula and workplace wellness programs targeting the reduction of metabolic disease burden [11].

To date, knowledge, attitude, and practice (KAP) research on MetS has primarily focused on patients with diabetes, those already diagnosed with MetS, or general population cohorts [10]. Robust evidence pertaining to ambulance support personnel, despite their heightened occupational exposure to metabolic risk factors, is conspicuously absent [18]. Evaluating their awareness, attitudes, and daily health practices could yield actionable insights into occupational health needs and inform the development of targeted interventions [19].

Workplace health promotion programs have delivered measurable improvements in lifestyle behaviors and cardiometabolic risk profiles across various occupational settings. Evidence-based strategies combining health education, systematic screening, physical activity promotion, nutritional counselling, and stress management training can yield substantial disease prevention benefits. However, successful program design necessitates a thorough understanding of the existing knowledge, attitude, and practice profile of the target workforce [20]. This study aims to assess knowledge, attitude, and practice (KAP) regarding MetS among ambulance support personnel to identify awareness gaps and inform targeted occupational health interventions.

## Materials and methods

This cross-sectional observational study was conducted in Karad City, Maharashtra, to evaluate knowledge, attitude, and preventive practices regarding metabolic syndrome among ambulance support personnel. The Krishna Vishwa Vidyapeeth Institutional Ethics Committee issued approval KVV/IEC/03/2026. A total of 119 personnel actively engaged in ambulance services were enrolled. Participants were recruited from government and private ambulance services operating in Karad City using a convenience sampling method. The study did not include all ambulance service providers in the city but only those who met the eligibility criteria and were available during the study period. The study population comprised ambulance drivers, emergency medical technicians (EMTs), paramedics, and ancillary support staff involved in pre-hospital emergency care. Participants were recruited using a convenience sampling approach guided by predefined eligibility criteria. Convenience sampling was adopted because ambulance support personnel work in rotating shifts and emergency-based schedules, making probability-based recruitment difficult. This approach facilitated access to eligible participants during the study period. Data collection employed a structured questionnaire designed to evaluate KAP domains relevant to metabolic syndrome. Data collection employed a structured questionnaire designed to evaluate KAP domains relevant to MetS. The collected data were subsequently analyzed to determine awareness levels and behavioral health profiles among the sample.

Inclusion and exclusion criteria

Eligibility criteria required participants to be ambulance support personnel aged between 30 and 50 years, including ambulance drivers, EMTs, paramedics, and related support staff, with a minimum of six months of professional experience in pre-hospital emergency care. Participants were required to be willing to participate in the study and provide informed consent before enrolment. Individuals with severe medical conditions, those who had undergone recent metabolic health training, and those receiving medications known to influence metabolic parameters were excluded to minimize potential confounding.

Data source and tools

Data were collected using a structured Google Form (Google, Inc., Mountain View, CA) questionnaire developed after an extensive review of the literature on metabolic syndrome and previously published knowledge, attitude, and practice (KAP) studies. The questionnaire consisted of four sections. Section I collected demographic information, including age, gender, designation, work experience, and educational background. Section II assessed knowledge regarding MetS and included eight items related to risk factors, symptoms, complications, and preventive measures. Section III evaluated attitudes toward MetS prevention and management and consisted of six items. Section IV assessed preventive health practices and included 10 items related to physical activity, dietary habits, health monitoring, stress management, and sleep hygiene. Participation was voluntary, and informed consent was obtained electronically before questionnaire completion. All responses were collected anonymously and maintained confidentially throughout the study. No formal pilot testing, content validation, or reliability assessment (e.g., Cronbach’s alpha) was conducted prior to data collection. The complete questionnaire used for data collection is provided in the Appendices.

Outcome measures

The structured questionnaire served as the primary outcome measure and consisted of four sections.

Knowledge scores ranged from 0 to 8 and were categorized as poor (0-3), moderate (4-6), and good (7-8). Attitude scores ranged from 0 to 6 and were categorized as negative (0-2), neutral (3-4), and positive (5-6). Practice scores ranged from 0 to 10 and were categorized as poor (0-4), fair (5-7), and good (8-10). These classifications were used to facilitate the interpretation of participants’ KAP levels regarding MetS.

Statistical analysis

A total of 119 ambulance support personnel were approached for participation, and all 119 completed the questionnaire, resulting in a response rate of 100%. Data were analyzed using descriptive statistical methods and presented as frequencies, percentages, means, and standard deviations. As the available dataset consisted of summarized responses rather than participant-level data, inferential statistical analyses were not performed.

## Results

Demographic and professional characteristics

A total of 119 ambulance support personnel completed the survey. Among these, 104 (87.4%) were men, and 15 (12.6%) were women. The predominant age group was 36-40 years. Regarding knowledge, 69 participants (58.0%) demonstrated a moderate level, and 39 (32.8%) demonstrated a good level. Attitudinal assessment revealed that 80 participants (67.2%) held a positive orientation toward MetS prevention and management. With respect to preventive practices, 72 participants (60.5%) achieved fair practice scores, while only 22 (18.5%) demonstrated good practices, indicating that applied health behaviors lagged behind knowledge and attitudinal levels.

Table 1 presents the demographic characteristics of the study participants. Of the 119 ambulance support personnel, 104 (87.4%) were men, and 15 (12.6%) were women. The majority of participants belonged to the 36-40 years age group (28.6%), followed by 30-35 years (26.9%), 41-45 years (24.4%), and 46-50 years (20.1%).

**Table 1:** Participant Demographics

Variable	Category	Frequency (n)	Percentage (%)
Gender	Male	104	87.4
	Female	15	12.6
Age group (years)	30-35	32	26.9
	36-40	34	28.6
	41-45	29	24.4
	46-50	24	20.1

Table 2 presents the professional characteristics of the study participants. Among the 119 ambulance support personnel, ambulance drivers constituted the largest group (50.4%), followed by EMTs (29.4%), paramedics (18.5%), and others (1.7%). Regarding work experience, the majority of participants had 1-5 years of experience (45.4%), followed by 6-10 years (29.4%), more than 10 years (15.1%), and less than one year (10.1%).

**Table 2:** Professional Characteristics EMT: emergency medical technician

Variable	Category	Frequency (n)	Percentage (%)
Designation	Ambulance driver	60	50.4
	EMT	35	29.4
	Paramedic	22	18.5
	Others	2	1.7
Work experience	Less than one year	12	10.1
	1-5 years	54	45.4
	6-10 years	35	29.4
	More than 10 years	18	15.1

Knowledge regarding metabolic syndrome

Table 3 presents the knowledge levels regarding metabolic syndrome among the participants. The majority of participants demonstrated a moderate level of knowledge (58.0%), followed by a good level of knowledge (32.8%), while 9.2% of participants had poor knowledge regarding metabolic syndrome.

**Table 3:** Knowledge Regarding Metabolic Syndrome (MetS) The majority of participants demonstrated a moderate level of knowledge regarding MetS (58.0%), followed by good knowledge (32.8%). Only 9.2% of participants demonstrated poor knowledge

Knowledge Level	Frequency (n)	Percentage (%)
Poor (0-3)	11	9.2
Moderate (4-6)	69	58
Good (7-8)	39	32.8
Total	119	100

Attitude regarding metabolic syndrome

Table 4 presents the attitude of participants regarding metabolic syndrome. Most participants exhibited a positive attitude (67.2%), while 26.1% showed a neutral attitude, and 6.7% demonstrated a negative attitude toward metabolic syndrome.

**Table 4:** Attitude Regarding Metabolic Syndrome (MetS) Most participants demonstrated a positive attitude toward MetS prevention and management (67.2%), while 26.1% exhibited a neutral attitude, and 6.7% exhibited a negative attitude

Attitude Level	Frequency (n)	Percentage (%)
Negative (0-2)	8	6.7
Neutral (3-4)	31	26.1
Positive (5-6)	80	67.2
Total	119	100

Practice regarding metabolic syndrome

Table 5 presents the practice levels regarding MetS among the participants. The majority of participants demonstrated fair practices (60.5%), while 21.0% exhibited poor practices, and 18.5% showed good practices related to the prevention and management of MetS.

**Table 5:** Practice Regarding Metabolic Syndrome The majority of participants demonstrated fair preventive practices (60.5%), while 21.0% demonstrated poor practices, and only 18.5% demonstrated good preventive practices

Practice Level	Frequency (n)	Percentage (%)
Poor (0-4)	25	21
Fair (5-7)	72	60.5
Good (8-10)	22	18.5
Total	119	100

Mean KAP scores

Table 6 presents the mean scores for knowledge, attitude, and practice regarding MetS among the participants. The mean knowledge score was 5.8 ± 1.4, the mean attitude score was 4.9 ± 1.1, and the mean practice score was 5.7 ± 1.8, indicating overall moderate levels of knowledge, attitude, and practice regarding MetS.

**Table 6:** Mean Knowledge, Attitude, and Practice Score The mean knowledge score was 5.8 ± 1.4, the mean attitude score was 4.9 ± 1.1, and the mean practice score was 5.7 ± 1.8, indicating overall moderate levels of knowledge, attitude, and practice regarding MetS among ambulance support personnel

Variable	Mean ± SD
Knowledge score	5.8 ± 1.4
Attitude score	4.9 ± 1.1
Practice score	5.7 ± 1.8

Interpretation

A total of 119 ambulance support personnel participated in the study. The majority of participants were men (87.4%), while women constituted 12.6% of the study population. Most participants belonged to the age group of 36-40 years and had 1-5 years of work experience in ambulance services.

The findings revealed that 69 participants (58.0%) demonstrated moderate knowledge regarding MetS, while 39 participants (32.8%) had good knowledge. Participants were generally aware of the major risk factors associated with metabolic syndrome, including obesity, hypertension, diabetes mellitus, physical inactivity, and unhealthy dietary habits.

Regarding attitude, 80 participants (67.2%) exhibited a positive attitude toward the prevention and management of MetS. Most participants acknowledged the importance of regular health checkups, physical activity, healthy dietary practices, and lifestyle modification in reducing the risk of MetS.

The assessment of preventive practices showed that 72 participants (60.5%) demonstrated fair practices, while only 22 participants (18.5%) exhibited good preventive practices. Although awareness regarding metabolic syndrome was satisfactory, the implementation of healthy lifestyle behaviors was comparatively lower.

The mean knowledge score was 5.8 ± 1.4, the mean attitude score was 4.9 ± 1.1, and the mean practice score was 5.7 ± 1.8. These findings indicate that participants possessed relatively good knowledge and positive attitudes; however, preventive practices were less satisfactory.

The results suggest the presence of a knowledge-practice gap among ambulance support personnel. Occupational factors such as long working hours, shift duties, irregular meal schedules, inadequate sleep, and work-related stress may contribute to the reduced adoption of healthy lifestyle practices despite adequate awareness regarding MetS.

## Discussion

This study characterizes the knowledge, attitude, and preventive practices regarding MetS among ambulance support personnel.

The demographic profile revealed a pronounced male predominance (87.4%), with the majority of participants in the 36-40-year age bracket and a substantial proportion having one to five years of professional experience. The demanding operational environment of ambulance services, featuring irregular shift patterns, high-acuity emergency duties, and sustained psychological stress, likely elevates the cardiometabolic risk exposure of this workforce [13,19].

Knowledge assessment demonstrated that 58% of participants possessed a moderate level of awareness, while 32.8% exhibited good knowledge of MetS. Participants broadly recognized the principal risk factors, including excess body weight, elevated blood pressure (BP), diabetes mellitus, sedentary behavior, poor dietary quality, and hereditary predisposition. These findings are consistent with comparable investigations among healthcare worker populations documenting the satisfactory foundational awareness of metabolic health and cardiovascular risk [10-12]. The predominance of moderate rather than high knowledge levels may reflect limited exposure to structured workplace health education programs and a greater occupational focus on emergency patient care than on personal health promotion. This finding suggests the need for regular educational initiatives to improve the awareness of MetS and its prevention among ambulance support personnel.

Attitudinal findings showed that 67.2% of participants had a positive orientation toward MetS prevention and management. Respondents widely acknowledged the protective value of regular exercise, balanced nutrition, periodic health monitoring, and behavioral modification. This pattern of favorable attitudes aligns with findings from prior KAP studies in related populations, suggesting that ambulance personnel are receptive to health promotion messaging even under demanding occupational conditions [10-12].

A key finding was the gap between knowledge and practice. Approximately 60.5% of participants achieved only fair practice scores, while only 18.5% demonstrated good preventive behaviors. Despite possessing reasonable awareness and constructive attitudes, a substantial proportion of respondents reported difficulties sustaining healthy lifestyle behaviors, including regular physical activity, dietary discipline, restorative sleep, and systematic health monitoring. This pattern closely parallels knowledge-practice gaps documented in prior studies examining lifestyle-related chronic disease in healthcare settings [11,12].

Mean scores further illustrated this imbalance: knowledge at 5.8 ± 1.4, attitude at 4.9 ± 1.1, and practice at 5.7 ± 1.8. Although knowledge and attitude scores indicate a reasonably informed and motivated workforce, the practice score underscores the persistent challenge of translating favorable dispositions into sustained behavioral change. This knowledge-practice disconnect represents the central actionable finding of the study.

Several occupation-specific factors plausibly account for this discrepancy. Ambulance staff routinely manage extended shift durations, respond to time-critical emergencies, cope with limited and unpredictable access to nutritious meals, and operate under persistent physical and psychological strain. These conditions may restrict opportunities for structured exercise, healthy dietary choices, adequate rest, and routine preventive healthcare. Prior research has consistently demonstrated that shift-based employment, sleep restriction, and occupational stress are independently associated with increased MetS risk among healthcare workers [13-17].

The overall pattern of findings is consistent with the broader literature indicating that healthcare professionals frequently demonstrate satisfactory awareness of non-communicable disease risk while exhibiting comparatively poor adherence to preventive lifestyle behaviors [10-12]. Occupational demands and workplace stressors have been repeatedly identified as primary barriers to health behavior maintenance in clinical workforces [13-20].

This study has several limitations. The cross-sectional design precludes causal inference, and the use of convenience sampling from a single city may limit the generalizability of the findings. The questionnaire was developed based on the available literature but was not subjected to formal pilot testing, content validation, or reliability assessment. In addition, the findings were based on self-reported responses, which may be influenced by recall and social desirability bias. Although this study primarily employed descriptive statistics to summarize participants’ knowledge, attitude, and practice regarding metabolic syndrome, inferential statistical analyses examining associations between participant characteristics and KAP scores could not be performed because participant-level response data were not retained after data summarization. Future multicenter studies using validated questionnaires, retaining participant-level data, and incorporating inferential statistical analyses with longitudinal follow-up are recommended to strengthen the evidence base regarding metabolic health behaviors among ambulance support personnel.

Collectively, these findings highlight the need for systematically delivered, context-sensitive interventions targeting ambulance support personnel. Evidence-based strategies that combine health literacy enhancement, accessible workplace wellness resources, organizational support for physical activity, nutritional guidance, and structured stress management programming hold significant promise for improving practice-level outcomes and reducing the aggregate metabolic disease burden in this occupational group.

## Conclusions

The present study assessed knowledge, attitude, and preventive health practices regarding MetS among ambulance support personnel in Karad City, Maharashtra. Findings indicate that the majority of participants possessed moderate knowledge and favorable attitudes toward MetS and its associated risk factors. Participants broadly recognized the protective roles of regular physical activity, balanced nutrition, periodic clinical checkups, and body weight management. Notwithstanding adequate knowledge and positive attitudes, the adoption of preventive health behaviors was comparatively limited, with a considerable proportion of participants achieving only fair practice scores. This gap between awareness and behavioral action likely reflects the influence of occupational variables intrinsic to ambulance work, including protracted duty hours, shift rotation, erratic eating patterns, physical fatigue, and cumulative occupational stress, all of which impede the consistent enactment of health-protective behaviors.

These findings affirm that awareness, while necessary, is insufficient to drive meaningful and sustained behavioral change. Workplace-based interventions addressing health education, lifestyle modification support, stress resilience building, and regular metabolic health screening are therefore essential. Occupational health programs should actively cultivate preventive behaviors and ensure equitable access to resources that support the physical and metabolic well-being of this workforce. In summary, targeted workplace wellness strategies are urgently needed to reduce the risk of MetS and enhance the long-term health and quality of life of ambulance support personnel.

## Appendices

Questionnaire

Section A: Sociodemographic and Health Profile

 Age (years): __________________________

Gender: ☐ Male ☐ Female ☐ Other

Designation: ☐ Ambulance driver ☐ EMT ☐ Paramedic ☐ Other

Years of work experience: ☐ <1 ☐ 1-5 ☐ 6-10 ☐ >10

Height (cm): __________________________

Weight (kg): __________________________

Do you have any chronic illness (≥3 months)? ☐ Yes ☐ No

If yes, specify: ☐ Diabetes ☐ Hypertension ☐ Dyslipidemia ☐ Other

Family history of diabetes/hypertension/heart disease? ☐ Yes ☐ No

Section B: Knowledge About Metabolic Syndrome

1. Metabolic syndrome is a cluster of metabolic risk factors. ☐ Yes ☐ No

2. Central (abdominal) obesity is a component of metabolic syndrome. ☐ Yes ☐ No

3. High blood pressure is related to metabolic syndrome. ☐ Yes ☐ No

4. High blood sugar levels increase the risk of metabolic syndrome. ☐ Yes ☐ No

5. A sedentary lifestyle increases the risk of metabolic syndrome. ☐ Yes ☐ No

6. Metabolic syndrome increases the risk of heart disease and stroke. ☐ Yes ☐ No

7. Lifestyle modification can prevent metabolic syndrome. ☐ Yes ☐ No

8. Long working hours and night shifts increase the risk of metabolic syndrome. ☐ Yes ☐ No

Section C: Attitude Toward Metabolic Syndrome

9. Metabolic syndrome is a serious health condition. ☐ Yes ☐ No

10. Ambulance support personnel are at higher risk of metabolic syndrome. ☐ Yes ☐ No

11. Regular health screening is important even without symptoms. ☐ Yes ☐ No

12. Maintaining a healthy lifestyle is important despite work pressure. ☐ Yes ☐ No

13. I am concerned about my future risk of metabolic syndrome. ☐ Yes ☐ No

14. Prevention of metabolic syndrome is necessary for long-term health. ☐ Yes ☐ No

Section D: Practices Related to Metabolic Syndrome

15. I perform regular physical activity. ☐ Yes ☐ No

16. I consume fruits and vegetables regularly. ☐ Yes ☐ No

17. I avoid frequent fast food and packaged food consumption. ☐ Yes ☐ No

18. I avoid skipping meals due to duty schedules. ☐ Yes ☐ No

19. I avoid eating late at night after duty hours. ☐ Yes ☐ No

20. I get adequate sleep daily. ☐ Yes ☐ No

21. I do not smoke or use tobacco products. ☐ Yes ☐ No

22. I limit alcohol consumption. ☐ Yes ☐ No

23. I undergo regular health checkups for BP, sugar, and cholesterol. ☐ Yes ☐ No

24. I manage work-related stress using healthy methods. ☐ Yes ☐ No

Scoring and Interpretation

Scoring system • Yes = 1 point • No = 0 point Knowledge score (0-8) 0-3 = Poor knowledge 4-6 = Moderate knowledge 7-8 = Good knowledge Attitude score (0-6) 0-2 = Negative attitude 3-4 = Neutral attitude 5-6 = Positive attitude Practice score (0-10) 0-4 = Poor practice 5-7 = Fair practice 8-10 = Good practice Higher scores indicate better knowledge, a positive attitude, and healthier practices regarding metabolic syndrome.
